# Years of life lost: A call to achieve equitable end-of-life care among children

**DOI:** 10.34172/hpp.2022.45

**Published:** 2022-12-31

**Authors:** Aysha Jawed

**Affiliations:** Department of Pediatric and OB/GYN Social Work, Johns Hopkins Children’s Center, Baltimore, MD, USA

**Keywords:** Pediatric, Hospice, Health equity, Palliative care, Life limiting illness, End-of-life

## Abstract

Significant disparities continue to exist in access to inpatient pediatric hospice care among children at the end-of-life. Increasingly more children at this stage are dying in the hospital or at home on hospice which is not always an acceptable option to the children and their families. Two clinical case examples illustrate implementation of these options in practice. A missing link exists in healthcare systems across developed and developing countries in pediatric end-of-life care. Currently, the primary options involve selecting between hospital and home-based hospice care. Proposing to increase access to inpatient pediatric hospice services could potentially increase acceptability of this option to honor the child in line with the family’s preferences, goals, wishes, and values. In addition, inpatient pediatric hospice could offset costs from preventable hospitalizations and overall high-cost healthcare utilization. Oftentimes, readmissions impact decision-making among caregivers that include changes in code status from Do Not Resuscitate/Do Not Intubate (DNR/DNI) to full curative care, thereby resulting in medicalization or overmedicalization of the child. It follows that reduced healthcare expenditures will increase cost efficiency across the healthcare system. Achieving health equity in palliative care among adult and pediatric patients at the end-of-life is a longstanding goal of the World Health Organization (WHO) and the United Nations International Children’s Emergency Fund (UNICEF). Proposing to mitigate disparities in palliative care among children through inpatient hospice as another viable option for their families could contribute to the larger overarching goal of achieving health equity in end-of-life care across the world.

## Introduction

 Pediatric hospice programs are oriented towards providing compassionate and comprehensive palliative care as well as psychosocial and socioemotional support to children with life-limiting illnesses at the end-of-life and their families.^1^ These programs can provide hospice care within a child’s home or in an inpatient setting (e.g. hospice facility/house, inpatient unit of an acute care hospital). Currently in our healthcare system, many pediatric hospice programs primarily provide end-of-life care in a child’s home. Several of these programs are part of adult hospice organizations and much fewer are freestanding pediatric-based programs.^2^

 In the United States, nearly 500 000 children have a life-limiting illness and are eligible for hospice.^3^ Almost 45 000 infants, children, and adolescents die in the U.S. every year.^4^ The National Hospice and Palliative Care Organization’s (NHPCO’s) most recent data uncovered that hospice services reach only about 10% of children who die in the U.S.^3,5^ Further, majority of these children were enrolled in hospice through adult organizations.^2^ 75% of these adult hospice agencies serve pediatric patients. However, most of them serve fewer than 10 children per year and further only a small proportion have dedicated pediatric programs.^2^ In addition, there are children across the US that reside in resource limited settings which do not have access to pediatric hospice care.

 The NHPCO also uncovered through a national survey administered to hospices across the U.S. that 78% of them reported care provided to pediatric patients. However, majority of these hospice agencies did not have a formal pediatric program for these patients which corroborates more recent findings aforementioned from the NHPCO.^2^ Most recent data from the National Summary of Hospice Care revealed that only 14% of hospice programs that responded had formally established pediatric palliative care services inclusive of a multidisciplinary team specialized in pediatric end-of-life care.^2^

 As a direct consequence, although most children at the end-of-life continue to die at home, increasingly more children in this population are also dying in hospital settings.^6,7^ Many of these children have chronic, progressive, and degenerative conditions.^7^ A significant part of their care at the end-of-life involves keeping them safe and sound as well as optimizing pain and symptom management amidst maximizing patient-and-family-centered care.

## Limitations in the infrastructure of the healthcare system

 The landscape of the built hospital environment seeks to promote healing, not death. One of the gaps in our healthcare system impacts fragile children with life-limiting illness. There is a missing link between hospital and home-based palliative care that is ultimately resulting in a growing number of children dying across hospitals around the world. Not every family is comfortable having children pass away at home. The memory of the child dying at home and oftentimes the space in the home where the child died is excruciatingly painful. Intrusive and distressing thoughts and emotions persist that make it harder for some families to continue living in their home knowing that the child died there.

 Pediatric inpatient hospice programs are a much-needed resource across the world but unfortunately, there is a significant scarcity of them.^8^ There are many reasons for this scarcity – existing hospice policies, staffing, health insurance status, and over emphasis on home-based hospice. Of note, there are only three specific facilities in the U.S. designed for children at the end-of-life. These houses are located in the Midwest (Minneapolis, Minnesota) and the West Coast (San Leandro, California and Phoenix, Arizona).^9^

 Based on existing data, England has an abundance of these resources with currently 41 hospice facilities specifically designed for children.^9^ Australia houses one pediatric inpatient hospice, the Bear Cottage that is affiliated with the Sydney Children’s Hospital Networks.^10^ Japan follows the pediatric hospice model in England and created its first inpatient hospice home, Momiji House in Tokyo that is operated by a healthcare institution and provides both end-of-life and respite care for children with life-limiting conditions and their families.^11^

 End-of-life among children is a growing area of research and practice.^6,7,8^ The reality is that the resources to support children and their families during this critical point in time are not enough.^8^ Many adult hospice agencies are well-staffed with nursing, respiratory therapists, physicians, social workers and more frontline staff to care for adult patients while they are admitted in hospice facilities. Unfortunately, there is a significant gradient with respect to the inpatient hospice resources accessible to children at the end-of-life and their families.

## Longevity considerations at the end-of-life

 The end-of-life stage for every patient is unique and different. Patients can sometimes be in this stage for months to years. During this time, it can become increasingly exhausting for their caregivers to provide supportive care around the clock. Given that many more adult hospice programs have inpatient units that are up and running, they can provide inpatient respite care for a certain number of days nearly each month to relieve caregivers. Unfortunately, there are significant limitations in access to inpatient pediatric hospice care in the U.S. and around the world.^8^ This reality further increases the number of children either dying in the hospital or at home when it is not always acceptable to the family; however, they are bound by helplessness in decision-making with the limited options.

## Goals of care implications in medical decision-making

 As a pediatric social worker at a large academic institution inclusive of both adult and pediatric centers, I have supported many patients who presented for care at the end-of-life. Some of these patients were well-known over months or years, returning for care while enrolled in home-based hospice. A frequently cited reason for readmission among several caregivers of these children was fluctuations in their decision-making when they perceived that their child is moments away from death. In that instance, they have reported a range of complex and distressing emotions centered on not desiring to have their child die at home which ultimately precipitated bringing their child to the hospital. Similarly in an article from the New York Times that explored existing inpatient pediatric hospice programs that predominate in the U.S. and England, preferences of children and their families were to find a location for the child to die outside the home or hospital environment.^9^

 At my academic institution, we have seen several children remain hospitalized until they take their last breath. We have also seen caregivers have a change of heart and reverse resuscitation and intubation status from Do Not Resuscitate/Do Not Intubate (DNR/DNI) to full code warranting full curative treatment for their child. This certainly raises ethical questions about whether medicalizing or overmedicalizing the child at this stage is in the best interest of the child and further could prolong suffering and inevitable mortality in the context of their already compromised quality of life.

## End-of-life clinical case examples

 Of note, a handful of these children in my healthcare institution presented from different developing countries around the world. Two cases that will likely stay with me throughout my clinical practice involve a patient with osteogenesis imperfecta (OI) type 8 and another baby with short gut syndrome. In both cases, parents were well-aware of their child’s debilitating prognosis. Also in both instances, parents were informed that they had two options: either prepare for the child to die in the hospital or have the child discharged to home-based hospice. One mother from Ghana decided to have her infant with OI type 8 remain hospitalized for nearly two weeks and observed significant decompensation in her child until time of death. In the second instance, parents from El Salvador decided to take their child with short gut syndrome home and celebrate her with their friends, family, pastor as well as members of their church community along with support from hospice. Their goal was to fill her last moments in life with love and honor. Both examples exemplify that there is a gap with respect to the range of end-of-life care options provided to a family since inpatient pediatric hospice programs were not an accessible option given their nonexistence in the State of Maryland – the only viable options were in-home hospice or dying in the hospital.

## Operational factors and capacity building for inpatient pediatric hospice care

###  Existing state of staffing capacity 

 One of the primary reasons that makes it challenging to operate pediatric hospice facilities is the significant shortage of nursing staff to care for these patients during this time. Many nurses are not well-trained in pediatric palliative patient care.^8^ For example, one of our pediatric hospice programs in the State of Maryland has a built pediatric inpatient unit in existence for greater than two years. However to date, it has not been staffed given scarcity of pediatric nurses. In turn, no patients have had opportunities to stay there during the end-of-life. For this reason, the hospice agency can only provide home-based respite for children.

 In one study that surveyed 551 hospice nurses across three states in the U.S (Tennessee, Mississippi, and Arkansas), almost 90% of them reported training in pediatric palliative care and further 50% had no pediatric hospice experience.^3^ In addition, greater than 70% of nurses reported limited experience in pediatric pain and symptom management for actively dying children.^3^ Findings from another study in Georgia revealed similar findings with only 50% of hospice nurses reporting previous experience and practice in caring for children at the end-of-life.^12^ The rest of the hospice nurses in this study also cited scarcity of training opportunities in this sphere.

 Staffing pediatric hospice facilities across developed and developing countries is crucial. It follows that increased staffing could subsequently inform inpatient pediatric hospice for consideration as a viable option and in turn increase acceptability of palliative care services in optimizing their child’s end-of-life. It is further imperative to train healthcare staff across the world in caring for these fragile patients during this vulnerable time in their lives. Empowering staff to feel confident in providing palliative care could also increase the likelihood of building a strengthened palliative care global workforce.

###  Cost efficiency across the healthcare system

 An additional consideration for supporting the development of inpatient pediatric hospice facilities across the world is to reduce healthcare expenditures stemming from preventable hospitalizations when home-based hospice is the only alternative viable option. In addition from my practice, changes in caregiver decision-making secondary to hospitalizations are prevalent and subsequently exert further strain on our already strained healthcare system. These decisions can involve medicalization or overmedicalization of the child. Provision of inpatient pediatric hospice as an option can offset these substantial costs.

###  Financial sustainability challenges

 Finances present a significant challenge in operating a pediatric hospice facility. In 2010, the Ryan House in Phoenix, Arizona was the second one to open in the U.S.^9^ However to achieve financial sustainability over time, it ultimately formed a partnership with an adult hospice facility and thereby could not operate as a freestanding pediatric hospice program.^9^ Similarly in 2011, a pediatric hospice facility, Dr. Bob’s Place opened in Baltimore, Maryland. However given depletion of funding sources, it ultimately closed down after two years.^9^

 Of note, many existing inpatient and home-based hospice programs require health insurance coverage for enrollment eligibility as well. Finding ways to ensure coverage for our fragile patients who are uninsured or underinsured through programs of the State such as Children’s Medical Services or grant-funded programs across state, national, and global organizations is critical in ensuring equity in palliative care among all patients with life-limiting illness.

 Breaking through the complexities in coverage within the bureaucratic structure of our tiered payment system is crucial. One strategy is to create a financial model in each country that supports and sustains pediatric inpatient hospice programs over time. Further endeavors could involve critically examining and identifying diverse licensures, regulations (e.g. building codes), and additional parameters across countries which can help establish a pathway in developing more inpatient pediatric hospice facilities.

###  Additional value-based measures in pediatric palliative care: acceptability and preferences

 One pediatric hospice facility, Crescent Cove in Minneapolis, Minnesota has received positive feedback from families of children who were actively dying.^9^ A terminally ill child’s caregiver described finding “security of the hospital” within the facility. A different family shared the same sentiment in the solace of healthcare in this facility that involved the intake of durable medical equipment and medical staffing around the clock. Another family described that Crescent Cove was a special place in peacefully achieving the end of life for their child.

 Crescent Cove along with the two remaining pediatric hospice facilities in the U.S. also present respite care options for families by giving them breaks from caring for their seriously ill children.^9^ Families feel well-supported knowing that their children are staying one or more nights under the 24-hour care of specialized nurses, assistants, and volunteers. In this pediatric hospice care model, respite care is recognized as a consistent element on a continuum from time of diagnosis to death.

 Taking case examples from my clinical practice along with qualitative information from existing pediatric inpatient pediatric hospice programs in the U.S., most families do not want their child to die in the hospital. However in many cases across the world, oftentimes the only other choice is home-based hospice care which in turn significantly affects their decision. It follows that inpatient pediatric hospice care could address this gap in care between hospital and in-home options.

###  Existing state of pediatric hospice policies and regulations 

 In England, the National Health Service and local governments are actively providing oversight and cover 22% of pediatric inpatient hospice costs.^9^ There are more inpatient resources available given regulatory standards for the government to be involved in the accessibility of these resources. It follows that more government support has increased funding for the 41 inpatient pediatric hospice programs in England. Existing measures also involve the implementation of an annual required Children’s Hospice Week to raise money for funding of these programs.^9^

 In the U.S., the eligibility criteria for hospice care among children is similar to the criteria in existence for Medicare beneficiaries. The existing hospice policies specifically require that patients must have a life-limiting diagnosis that is expected to have a prognosis of six months or less time.^13^ Additional policy parameter pertains to code status (Do Not Resuscitate). De-escalation in care is also prevalent across these policies. The Patient Protection and Affordable Care Act of 2010 presented a continued option for hospice care concurrent with curative therapies covered by Medicaid state health insurance for children with serious illness by increasing the maximum age range for coverage to 21 years.^13,14^

 Uganda has developed an integrated pediatric and adult palliative care policy to increase access to end-of-life care, palliative education, and access to pain medications.^15^ In Ireland prior to the development of policy on pediatric palliative care, a needs assessment was conducted which uncovered that approximately 1400 children were living with a life-limiting illness and among them, many could not access needed care.^16^ In turn, a national policy was developed to provide the vision and framework for palliative care services among children in Ireland.^17^ Lastly in Serbia, the Serbian Ministry of Health developed a national strategy and model of palliative care service delivery for both children and adults following findings uncovered in a needs assessment on pediatric palliative care.^18^

 In addition, a global online survey was conducted across 198 countries to assess for the presence and implementation of palliative care strategies, plans, legislation, and government resources.^19^ Findings uncovered were mixed and revealed substantial variation in degree of implementation of palliative care strategies across 55 countries. There were 47 countries that referenced palliative care in national law. Further, 24 countries have implemented some form of legislation on provision of palliative care and have integrated this content into their respective constitutions. Lastly, 66 countries have designated governmental responsibility for palliative care.

 Based on these findings, critical examination of our existing hospice policies for children across the world are warranted. Although children are eligible for hospice if they have a life-limiting diagnosis of six months or less to live, the range of options are not always acceptable and in line with the goals, wishes, values, preferences, and vision of their caregivers to honor their child during these last moments of life.

###  Clinical framework implications: patient and family-centered care

 An inpatient hospice environment is substantially different from a pediatric unit (e.g. medical/surgical, intensive care) in a hospital. An inpatient pediatric hospice unit presents a unique opportunity to promote an increasingly nurturing and peaceful environment to support the child and family at the end-of-life. Furthermore, it will not limit visitors surrounding the child during this time. Hospitals have limitations in visitation which have substantially increased during our pandemic era. Requesting to make an exception at the end-of-life in hospitals oftentimes is not feasible. It follows that visitation in inpatient pediatric hospice seeks to maximize patient-and-family-centered care.

## Health equity implications

 Integrating inpatient pediatric hospice into the healthcare system is essential towards achieving an equitable palliative care landscape for children and their families. It follows that addressing this gap in the healthcare system will seek towards achieving health equity by optimizing attainment in affordability, accessibility, and quality of palliative care to the highest standard for all patients across age, gender, socioeconomic, racial and ethnic groups. Furthermore, it will contribute towards the larger goal of both the World Health Organization (WHO) and the United Nations International Children’s Emergency Fund (UNICEF) to optimize the end-of-life for both palliative pediatric and adult patients. One prevalent issue with respect to increasing equity in palliative care across developing countries is limited or no access to pain medications (e.g. morphine) for patients at the end-of-life. Part of increasing health equity for patients at the end-of-life is to ensure that each of them has access to these medications as the basis to ameliorate suffering to the fullest extent possible. As is the case across adult inpatient hospice settings, the inception of more inpatient pediatric hospice programs will significantly increase accessibility and mobilization of resources in the care of pediatric palliative patients.

## Funding

 None.

## Ethical Approval

 Not applicable.

## Competing Interests

 The author has no competing interests to declare that are relevant to the content of this article.
